# Quantification of Ergonomic Exposures for Restaurant Servers

**DOI:** 10.4172/2165-7556.1000166

**Published:** 2016-01

**Authors:** Angela C Wills, Kermit G Davis, Susan E Kotowski

**Affiliations:** 1 Low Back Biomechanics and Workplace Stress Laboratory, University of Cincinnati, Cincinnati, Ohio, United States; 2 College of Allied Health Sciences, University of Cincinnati, Cincinnati, Ohio, United States

**Keywords:** Waitress, Lifting, Standing, Musculoskeletal disorders

## Abstract

Musculoskeletal disorders have the potential to impact a tremendous number of waitresses and waiters in the United States, yet very little is known about the ergonomic risk factors that these workers routinely encounter. The objective of the study was to document the potential ergonomic stressors that restaurant servers are exposed to during a typical shift. During a typical work shift, the following data was collected on twenty servers from three different restaurants: the weight and frequency of trays transferred as well as postures adopted when serving by direct observation; amount of time sitting, standing and walking by an ActivPal activity device; and workload perception and current discomforts by survey. Observations revealed that servers carried 16.4 kg per hour or 6.3 kg per tray. More than 90% of the servers reported spending between 5 and 8 hours standing during their shift. Objective measures confirmed spending a large amount of time on their feet (76% of time standing or walking). At the end of the shift, the body region with the greatest end of the shift discomfort was the upper back (55%), followed by the neck (45%) and lower back (50%) regions. In all, the current study provides a glimpse into the demands on the servers. All indications were the current results were lower than normal shifts. The bottom line was that the weight of the tray transferred, time spent standing and walking, and the awkward postures adopted by servers increase the risk of musculoskeletal disorders (MSDs). However, no direct link to MSDs can be drawn from the current study. In general, while the weight or number of trays served per hour was not particularly high, the load lifted represented a risk to the servers, especially when peak times require many more trays served.

## Introduction

In 2011, the total number of waitresses and waiters employed in the United States was 2.2 million [1]. Further, the number of servers is expected to grow by about 10% between 2006 and 2016 [1]. Full-service restaurants (e.g. sit down facilities) represent approximately 40% of all restaurants in the industry. As such, these full-service establishments employ about 900,000 servers [1].The typical worker has traditionally been young, low education and oftentimes in transitional employment.

While musculoskeletal disorders (MSDs) appear to be substantial for wait staff with incidence rate of 9.8 per 10,000 servers [2], little is known about the actual ergonomic stressors that servers are exposed to during their typical work shift. Through casual observation, one can see many factors present for servers that have traditionally been associated with MSDs such as amount of weight lifted and carried, awkward postures including forward or side bending, extended reaching, repetitive lifting of heavy and awkward loads, and prolonged standing.

The amount of weight that servers carry constantly changes with every order, but appears to be significant. A server can have just a soup and salad for one order, but the next order may be multiple entrée plates on a tray. Food can be carried by hand or by tray. While there has been no studies actually quantifying the weight that servers lift and carry, weight has been identified as a risk factor for low back injuries in other industries [3]. The critical value identified seems to be 11.4 kg for low back injuries [3–5]. In a study by Marras and associates [6], the average weight handled by high risk jobs was 11.4 kg as compared to 8.3 kg for low risk jobs. Further, Neumann et al. [7] reported increased risk for 12 kg weights. Bottom line, the amount of weight lifted is potential a risk factor for low back injuries and potentially shoulder injuries and needs to quantified.

Another potential important ergonomic risk factor for servers is task repetition. The servers are constantly serving plates and trays of food. However, the exact number of times a server lifts and carries of food, drinks, and trays is unknown. Based on previous research, repetitive lifting has been found to be associated with low back and shoulder injuries when lifting weight above 11.4 kg [5,8]. Ferguson and Marras [9] reported 62% of the studies that investigated lift rate reported a positive association with low back disorders.

Wait staff routinely face situations that require them to work around different obstacles such as reaching around individuals while serving, reaching across tables to place or pick up plates or glasses, or having to hold plates or glasses away from the body because multiple plates are staged on the server’s arm. These situations impact the moment arm between the low back to the object in the hand. Several previous studies focusing on material handing have found an increased risk of low back pain (LBP) as the horizontal distance increases Marras et al [6,10,11].

Servers are required to perform many different activities that may produce awkward postures: preparing for a meal to be brought out from the kitchen; reaching into the windows in the kitchen to get the plates of food; reaching for a tray jack; picking up and carrying the food; holding trays above the shoulders and head; placing the trays down on the jack or table; bending down to pick up the dishes of tray or table; and reaching across table or around people to serve their food. Flexion or extension of various body regions (e.g. trunk, shoulders, etc.) individually, or in combination with twisting are factors known to increase LBP [3,8,9,12–15]. However, actual postures during serving have yet to be quantified.

Serving jobs require long periods of being on your feet without frequent breaks. Two reasons for infrequent breaks are: 1) servers are not given the opportunity by management and 2) servers do not want to miss out on the potential opportunity to make money while on a break. Along with standing, severs are constantly walking around the restaurant assisting customers or performing serving duties. Walking and standing for long durations have been related to low back pain and lower extremity disorders. Researchers have identified prolonged standing and walking with many different negative health outcomes such as chronic venous insufficiency [16–19], varicose veins [20] symptom free venous reflux [21], hip osteoarthritis [22], and leg and foot pain [23]. A strong relationship has been found for prolonged occupational standing and low back pain [23–26]. As with the other potential risk factors, prolonged standing and long periods of being of their feet (e.g. walking) appear to plague servers on their jobs but there has been no actual measurement.

While there is ample support of the risk factors commonly seen for servers, less than a handful of studies exist which focus specifically on restaurant service staff. Dempsey and Filiaggi [27] studied the job demands among 100 servers in 10 casual dining areas using a cross-sectional survey provided to the servers. The survey documented the servers’ musculoskeletal discomfort attributed to work. The top two body regions suffering pain were low back (28%) and shoulder (11%) [27]. Jones and associates [28] examined the physical demands of occupational tasks in neighborhood pubs in British Columbia, Canada. These investigators looked at three specific tasks in relation to servers using RULA (Rapid Upper Limb Assessment): carrying task, tray lifting task and stoop-to-serve task, with all three tasks being deemed to be a significant risk to the servers based on RULA. Another group looked into training techniques that focused on safe tray carrying among cocktail servers [29]. However, it appeared that training was not a viable intervention for servers.

From the summary of the limited studies that have investigated the potential risk factors that servers may be exposed to while serving customers as well as casual observation, there is a need for direct quantification of the ergonomic risk factors for servers. The objective of this research study was to determine the exposure to ergonomic risk factors for servers (waitresses and waiters) in restaurant settings. Additionally, the study will document the discomfort that resulted from a single shift.

## Materials and Methods

### Study Overview

A research team observed and measured the ergonomic stressors for servers at three different restaurant locations in a Midwest metropolitan area. An entire shift was observed for each server as they performed their normal job duties. Ergonomic stressors were directly assessed by either observation (e.g. postural demands, frequency of events, etc) or quantitative measures such as weight of the trays being transferred to and from the tables. Two surveys were completed by the servers to document current perceived demands and discomfort symptoms in specific body regions.

### Study Sites

All three of the restaurant locations specialized in their own style of cuisine. They were all full-service restaurants and open 7 days a week. Restaurants 1 and 2 were privately owned while restaurant 3 was a franchise.

Restaurant 1 specialized in American-style bar food (burgers, wings, pretzels, salads, etc.). Restaurant 1 had a large semi-circular bar in the middle of the restaurant, a large kitchen area, and three dining locations. The restaurant had a total of 13 servers and 3 bartenders, where one server also worked as a bartender on certain nights. During a typical night shift 4 to 6 servers would be working, while afternoon shifts usually had 2 to 3 servers.

Restaurant 2 specialized in hamburgers (burgers and salads). Restaurant 2 had a small square bar in the middle of the restaurant, a large kitchen behind the bar, and a dining area that completely surrounded the bar area. This location had a total of 12 servers and 3 bartenders. The typical lunch shift would have 3 to 4 servers while dinner shifts had 5 to 6 servers.

Restaurant 3 specialized in Italian cuisine (pastas, pizza, calzones, salads, etc). Restaurant 3 had a large kitchen with a relatively small dining area and no bar. It also had a small area to serve take-out orders. This location employed 7 servers and no bartenders. A typical dinner shift had 5 severs while the typical lunch shift had 3 servers.

### Subjects

A total of 20 wait staff (5 males and 15 females) were recruited from one of the three restaurant settings in a large Midwest metropolitan area. Inclusion criteria for participation were the following: at least 2 or more years of experience serving (total), serving at current location for more than 6 months, no major surgeries within the last 2 years, not currently pregnant, and being older than the age of 18 (which was for convenience for consent but was not an issue given all three restaurants served alcohol and required servers to be at least 18 years old).

### Experimental Design

The design of the study was a cross-sectional observational study where one member of the research team quantified the stressors of each of the waitresses/waiters over their entire shift. At each of the restaurants, there were multiple shifts observed including day (lunch) and night (dinner) and a mix of both shifts. A typical day shift at all three locations started between 10:30-11:00 am and went till 2:00-3:00pm. The dinner shift started between 4-5pm and would last till 9-10 pm. The hour between 3-4 pm was designated for shift change, clean up and restocking of the plates, drinking cups and food. The breakdown of the observed shifts for this study includes 9 day shifts and 11 night shifts.

### Assessment Techniques

#### Observation worksheet

Frequency of tray carrying and postures of specific body joints were recorded by one of the researchers through data worksheets. The worksheet had checklist entries that recorded the number of times a tray was transferred, what was being carried, and poor postures for the neck, shoulders, hands and wrists, elbows, upper back, lower back, hips, knees and lower legs and feet. The types of poor postures were based on RULA categories [30]. The following adverse posture categories were used: Neck (flexion > 20o, lateral extension > 30o, extension > 30o, rotation > 70o); Trunk (flexion > 60o, rotation < 30o , lateral flexion > 15o, extension > 25o); Shoulder (forward flexion > 180o, abduction > 180o, external rotation > 90o); Elbow (flexion < 145o), Upper arms (flexion > 20o, extension > 90o); Lower arms (flexion < 60o or > 100o); Wrist (flexion and extension > 45o , radial/ulnar deviation > 30o). For each lift, carry, or transfer of the trays, a research team member marked down the body posture relating to each specific body region.

Additional information including observation of how each server carried trays of food or held the food with their hands was recorded. There were six categories of how the tray was carried: 1) in front of the server, 2) on the shoulder, 3) on the finger tips, 4) with a flat palm, 5) above the shoulder in the air, and 6) number of hands (e.g. using one hand versus two hands). For any served tray, there could have been a combination of any of the 6 categories at one time. For example, a server could carry the tray of food on their shoulder with a flat palm or on their fingertips with one hand with or without support of the second hand. Lastly, the type of tray jack used when setting down trays was recorded. There are four tray jack categories: 1) waist high tray jack, 2) mid-thigh high tray jack, 3) holding the tray while serving, and 4) placing the tray on a table surface.

Data were gathered about the number of plates/glasses on the tray. The categories of number of glasses were: 1) bar glasses which were full of liquid, 2) plastic cups filled with liquid and 3) empty glasses, ether plastic or glass. The categories for the number of plates handled were: 1) full plates of food, 2) empty plates of food and 3) plates that still had some food remaining on them. For each of the glass and plate categories, a total number was recorded for each tray handled.

#### Tray weight

The tray weight was measured by having the server step on a scale located in a convenient position near the kitchen. The weight of the server and tray was recorded and then converted into tray weight later by subtracting the weight of the server. This method allowed for quick measurement without compromising the food safety and quality.

#### Physical activity

The amount of time sitting, standing and walking was captured by a physical activity logging device known as ActivPALTM (PAL Technologies Limited, United Kingdom). The ActivPalTM device was placed on upper thigh of the server prior to the beginning of each observed shift. The ActivPALTM is lightweight (15 grams) and small (7 mm thick) rectangular device capable of acquiring activity for 7 days and downloaded to a personal computer for further processing. The ActivPALTM was used to document the amount of time spent sitting, walking and standing as well as number of steps taken and energy expenditure. The amount of time spent in the different categories was converted into percentage of time per hour while the number of steps taken was calculated per hour of each shift. The validity of ActivPALTM has been shown to be strong by Aminian and Hinkson [31], with correlations between ActivPalTM and actual observation to be above 99% (r=0.99 ± 0.01). These researchers also reported that there was no misclassification of leg fidgeting to actual walking steps.

#### Current symptom survey

Participants completed a Current Symptom Survey at the start and end of the observed shifts. The Current Symptom Survey assessed current discomfort in various body regions on a numeric analogue scale of 0 to 10; 0 being no pain experienced, 3 mild pain, 5 moderate pain, and 10 severe pain. The 9 body regions were neck, shoulders, elbows, hands/wrists, upper back, low back, hip, knees and lower legs, and feet. The numerical analogue discomfort scale has been widely utilized by many fields to document pain reliably and accurately for pain attributable to cancer, musculoskeletal, rheumatic, and surgical [32].

#### Workload perception survey

The Workload Perception Survey assessed whether the server felt the observed shift was typical of the work demands for their restaurant for the given day and shift. The survey was composed of 7 questions that ask about 1) how the typical shift was relative to the current one being observed, 2) how many people they serve per hour on a normal day, 3) how many tables they would normally serve, 4) how many hours a week do they work, 5) how long they believe they stand, 6) how often they get to take breaks, and 7) what days do they normally work in a week.

#### Experimental procedures

Upon arriving at each of the restaurant locations, the logistics of the study were explained verbally to all of the participants (on a one-on-one basis) by the Primary Investigator (PI). Once any questions or concerns were addressed, the consent process was completed (approved by the Institutional Review Board) that included reading and signing the consent form. Next, each participant completed a Current Symptom Survey and the ActivPALTM was attached to upper thigh (via of the participants which included two sided tape and a self-adhesive bandage wrap). The ActivPal device was worn for the entire duration of the shift. Next, body weight was measured on a digital scale and recorded (fully clothed and wearing shoes). At this point, the servers started their shift with the research team member observing their postures and measuring the loads of the trays being carried.. Shifts were observed based on each server’s normal working shifts and day which included all days of the week, peak and slow periods, and multiple restaurants. When the server had a tray of food that needed to be served, the individual stepped on the scale while holding the tray of food so the weight of the tray and body were recorded. The weight of the tray and food was determined by taking total weight and subtracting the body weight.

At the end of the observed shift, the ActivPalTM was removed from the subject with the ActivPalTM being placed in a horizontal position to signal the stop in recording of the physical activity. The data was later transferred onto a computer for subsequent analyses. Next, the subjects completed the second of the two Current Symptom Surveys and the Workload Perception Survey. After the final two surveys were collected, the subjects were paid a small token of appreciation ($20.00) for their participation.

#### Statistical Analysis

Descriptive statistical analysis (means, standard deviations, frequencies, etc.) was used to determine estimates of the exposures for servers. In general, summary tables provided means or frequencies of events for the different ergonomic stressors. Given the nature of the study was observational and exploratory with limited subjects, no statistical analyses were deemed appropriate. As a result, summary data of the observations were provided in table format.

## Results

### Overall Hours Collected and Demographics

The total amount of time the servers were observed was 68.9 hours. The demographic data are summarized in Table 1. Restaurant 1 was observed for 30.35 hours, restaurant 2 for 15.4 hours, and restaurant 3 for 23.15 hours. Of the 20 servers observed, there were 5 males (25%) and 15 females (75%) with 2 males and 6 females for restaurant 1, 2 males and 3 females for restaurant 2, and 1 male and 6 females for restaurant 3. The average age for all 20 servers was 26 years. For servers working at restaurant 1, the average age was 26.4 years, restaurant 2 was 23.8 year, and restaurant 3 was 27 years. The height and weight of the servers at the three restaurants were similar to that of a typical working population (Table 1).

Overall, the average length of experience was 4.4 years of total years in serving (Table 1). Restaurant 1 had slightly more years of experience (5 years) with restaurant 2 having less (3 years). Restaurant 3 had about the overall average with 4.7 years of experience in serving. The breakdown of shifts (Table 1) was the following: Lunch shift had 9 servers (45%) with restaurant 1 having 2 servers, restaurant 2 having 3 servers, and restaurant 3 having 4 servers and Dinner shift had 11 servers (55%) with restaurant 1 having 6 servers, restaurant 2 having 2 servers, and restaurant 3 having 3 servers.

### Workload perception

The results from the Workload Perception Survey indicated that only 40% of the servers felt the observed shift was a typical shift while the remaining 60% thought it was lighter than usual (40% lighter and 20% lightly lighter) (Table 2). The number of people typically served during a normal shift reported by the servers had 5% reporting 1 to 3 people, 25% reporting 7 to 9 people, 30% reporting 10-12 people, and 40% reporting 12 or more people. The number of tables served per hour was reported by 5% as 1 to 2 tables, 40% as 3 to 4 tables, 35% as 5 to 6 tables, and 20% as 9 or more tables per hour. The number of hours worked in a week was reported to be less than 20 hours a week by 10% of the servers with 50% of the servers being part time, 35% of servers being full time (40 hours per week) and 5% of the servers working more than 40 hours a week. A small percentage of servers (5%) reported standing more than 2 hours but less than 5 hours while 90% of severs reported standing more than 5 hours but less than 8 hours. About 5% of the servers actually felt they stood more than 8 hours. When asked about the perceptions of breaks, 10% indicated that they took a break once an hour, 40% indicated once a shift, 35% said whenever they wanted, and 15% never took a break.

Finally, when asked what days they normally work, 60% of the servers indicated that they mixed days, 5% said only weekends, 15% said only weekdays, 5% only mornings, and 15% only nights.

### Current symptoms before and after shift

**Figure 1:**
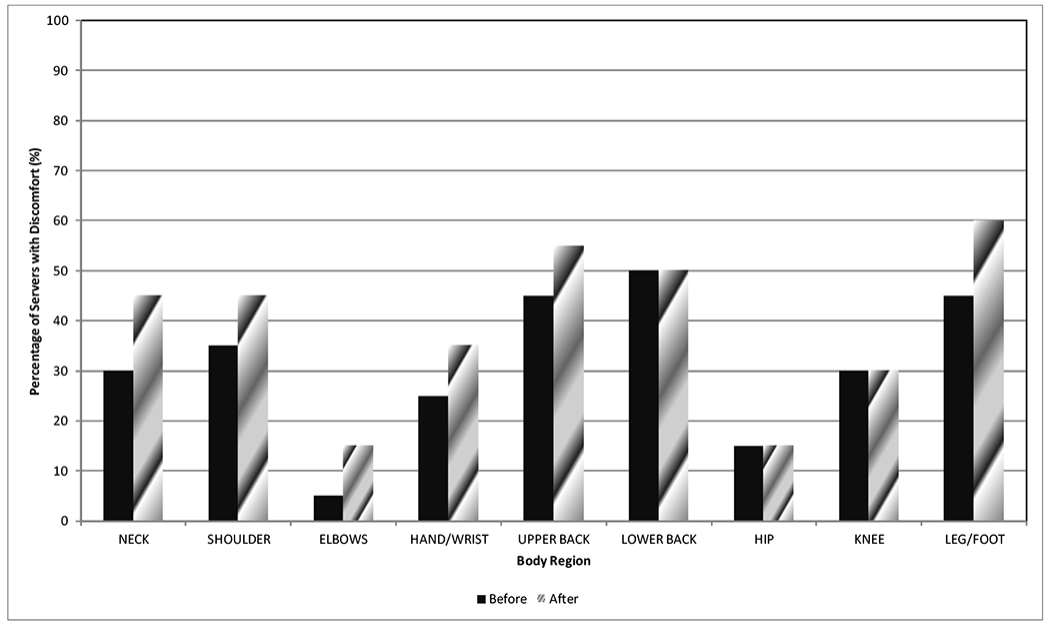
Percentage of servers in the study that had discomfort at the start and end of the shift being observed.

There was a general trend of increased pain at the end of the shift. The largest increase in pain for any of the body regions occurred in the upper back of 0.80 (55%). The other regions with increased pain at the end of the shift were neck (increased by 0.65 or 45%), lower back (increased by 0.60 or 50%), hand/wrist (increased by 0.45 or 35%), and leg/foot (increased by 0.40 or 60%). The body regions that had little increase in pain at the end of the shift were the hips with an increase of 0.20 (15%) and the elbows with an increase of 0.10 (15%). Two of the body regions actually had a decrease in pain between the end and start of the shift: shoulders had an average decrease of 0.10 (45%) and knees had a decrease of 0.05 (30%). Figures 1 shows how many of the body regions had a greater number of individuals with discomfort at the end of the shift than the start, specifically neck, shoulder, elbow, hand/wrist, upper back, and leg/foot.

### Weight lifted and transferred

The average amount of weight per hour lifted for all servers was 16.4 kg (see Table 3 for summary of the weights). While the average weight and total number of trays carried varied across the restaurants, the total weight per hour was 16.4 kg, average amount of weight per tray was 6.3 kg and number of tray carried per hour was 2.7. The total amount of weight per hour for lunch service was 16.3 kg in relation to the amount of weight per hour during the dinner service which was 16.6 kg. The amount of weight per tray for lunch service versus dinner service was 7.1kg and 5.8 kg, respectfully. Finally, the number of trays that were carried between the two shifts was 2.5 kg for the lunch shift and 2.9 kg for the dinner shift.

The averaged total amount of weight over shift for tray holding postures was 15.6 kg when using combination of transfers, 13.2 kg when transferring using fingers to support the tray, 16.8 kg when the tray is held in front of body, and 15.2 kg when transferring the tray on the shoulder. The amount of weight averaged per tray was: 5.5 kg for the combination transfer, 5.7 kg when using fingers technique to transfer the tray, 6.7 kg when the server held tray in front of body, and 6.8 kg when server carried the tray on the shoulder.

### Transfer technique and usage of tray jacks.

The frequency of the usage of the different tray transfer techniques is summarized in Table 4. For all 3 restaurants, there were a total of 133 tray transfers. There were 93 trays (69.9%) that were carried in front of the servers. On the other hand, the shoulder and hand flat techniques were used less frequent, 23 (17.3%) and 10 (7.5%), respectively. Finally, few transfers were observed to use the fingertip method (around 5% of observed transfers). During the lunch shift at all of the restaurants combined, 18% (24 times) of the servers carried the tray in front of themselves, 10.5% (14 times) on their shoulders, and 4.5% (6 times) on their fingertips and there were no servers who carried the tray with a flat palm. During the dinner service, 51.9% (69 times) of the servers carried the tray of food in front of their bodies, 6.8% (9 times) on their shoulders, 7.5% (10 times) carried the tray with a flat palm, and 0.8% (1 time) used their fingertips. Lunch and dinner servers seemed to prefer carrying the trays in front of their body as opposed to any other form of tray transfer. There were 4 different ways the trays could have been placed when serving the food. For all servers, there were a total of 124 tray jacks used.

### Transfer of Trays

Overall, the prevalence of carrying tray methods was 93 in front of server (23.25 trays/hour), 23 on the shoulders (65 adverse postures/hour), 10 flat palm (7.5 trays/hour), and 7 on their fingertips (5.3 trays/hour). During the lunch shift, there were a total of 24 (18.0%) times where the servers carried the tray in front of them, 14 (10.5%) carried the trays on their shoulders, 6 (4.5%) carried the trays of their fingertips, and no one carried them with a flat palm. During dinner shifts, it was observed that a total of 69 (51.9%) times where the server carried the trays in front of them, 9 (6.8%) times where the server carried the trays on their shoulders, 10 (7.5%) times where they carried trays with a flat palm and 1 (0.8%) time where a server used fingertips to carry the tray.

### Postures

Table 5 provides a summary of the awkward postures for the different body regions. A total of 260 poor postures were recorded during the observations. Several body regions were observed to have the greatest number of poor posture including elbow flexion, shoulder abduction, wrist extension, and neck side bending. For all three of the restaurants, there were a total of 85 observed instances of elbow flexion, the leading body region for poor postures: restaurants 1, 2 and 3 had 32, 20, and 33 awkward elbow postures, respectively. Neck side bending was the 2nd most occurring posture for all three restaurants with a total of 55 occurrences. Adverse shoulder abduction was observed a total of 30 incidences. Finally, another routinely observed poor posture occurred for wrist extension, which was observed a total of 29 times.

### Drinks carried

The total amount of drinks that were dropped off full and picked up empty was 571 glasses or 167.9 glasses per hour. The plastic glasses weighed 0.91 kg when full and 0.61 kg when empty. The bar glasses weighed 1.14 kg when full and 0.92 kg when empty. The total amount of full bar glasses was 178 (52.35 glasses/hour) for all three restaurants, with restaurants 1, 2, and 3 serving 99 glasses (26.05 glasses/hour), 73 glasses (23.54 glasses/hour) and 6 glasses (1.81 glasses/hour), respectively. The total number of plastics cups was 232 (68.23 glasses/hour) for all three restaurants.

### Physical activity

Servers at all three restaurants spent a total of 24.5% (25.5% per hour) sitting while being observed performing their tasks, 30.9% (13.9% per hour) of their time walking, while standing 44.6% (16.7% per hour) of the time, according to ActivPAL. The total number of steps per hour for all three restaurants was 609.8 steps. During the lunch service there were a total of 609.8 steps per hour and during dinner service there were 606.5 steps per hour. Restaurant 2 had the most total number of steps walked at 1,327.7 steps (690.3 steps per hour). The amount of energy expended during the working shifts was 1.5 or 0.5 MET per hour, dinner shifts had 0.1 MET per hour more than the lunch shift at 0.4 MET per hour, and the difference between all three restaurants was 0.1 MET per hour between restaurant 3 (0.6 MET per hour) and 1(0.5 MET per hour) or 2 (0.5 MET per hour).

## Discussion

The current study represents one of the few studies to investigate the ergonomic stressors for servers in restaurants. Previous research has concentrated on musculoskeletal symptoms for servers [27] and semi-quantitative whole body assessments like RULA [28]. The current study adopted an observational design where servers were followed through the entirety of their shift with objective measurement of weight carried on trays, frequency of carrying trays, and observed transfer technique, including awkward postures. The results showed that when servers transferred trays, the weight carried was significant, averaging 6.3 kg per tray. The amount of weight per tray represents about 73% of the average weight that has been reported for high risk jobs in industry [6]. Thus, the amount of weight lifted and carried routinely by servers potentially poses significant risk for the low back injuries and likely shoulder problems.

Overall, the number of trays carried by the servers was about 3 per hour, carrying 16.3 kg per hour. On the surface, this frequency seemed to be low but maybe more reflective that the observation dates were on slow activity shifts. More than half (60%) of the servers indicated that the observed days were less busy than their typical work days. As a result, the observed number of trays and weight carried may be significantly lower than typically seen for servers.

Servers spend a large percentage of their time on their feet, either standing (45%) or walking (31%). With most of the standing and walking occurring on hard floors either wood or concrete, long-term detrimental effects may occur in the lower extremities and lower back including: chronic venous insufficiency [16,17], varicose veins [18,19], low back pain [23,24] symptom free venous reflux [25], hip osteoarthritis [22], and leg and foot pain [24]. The perceptions also support a large portion of the time spent standing or walking with 90% of the servers indicating they stand between 5 and 8 hours per shift. The amount of walking translated into about 1000 steps per hour. During the observation, it is clear that the servers have frequent periods of motion where servers are constantly running around the restaurant getting drinks, taking orders, bringing out food, grabbing things (e.g. ketchup or other condiments), and cashing out the bill. Basically, the objective and subjective assessments of the standing and walking indicate a very dynamic and physical job.

One of the main mismatches between observed and preserved outcomes was for the amount of breaks. Based on the ActivPAL, servers sat down about 25% of the time which would be in direct contrast to the perceptions that indicated 55% of the servers had less than 1 break per shift. Limited breaks could further enhance the adverse effects of the high physical demands and being constantly on their feet.

The other main ergonomic risk would be how the trays are handled when delivering and returning food and dishes to the tables. The most widely used technique to transfer the trays was holding tray directly in front (70% of the trays transferred used the front technique). The other two commonly observed techniques were on the shoulder (17%) and holding hand flat (7.5%). The placement of the tray at the table also plays a role in the demands on the server. In general, servers set the trays the majority of the time on a table (63%) or on a tray jack (17%). Only a small portion of the trays were held throughout the serving (20%). The use of trays or tables would certainly minimize the physical demands on the servers with respect to holding the weight of the tray, food and dishes.

There may be several reasons why a server used a table instead of the tray jacks: 1) table was more stable when setting down a tray full of food; 2) the server was unable to grab a tray jack; and 3) transferring the trays in front of them would make it impossible to set-up a tray jack. Handling of the trays with food and/or dishes as well as other activities that the servers routinely did appeared to produce awkward postures in many of the body regions. Poor elbow postures were the most prevalent (33% of the awkward postures) with other regions of concern being neck (side bending-21%), wrist extension (11%), and shoulder abduction (12%). Many of these observed awkward postures were potentially linked to carrying and placing the trays, specifically the technique utilized. Overall, the frequency of the awkward postures were surprising low over the observed shifts, indicating that the weight being transferred is probably a larger issue than the postures adopted when transferring the trays and food, although the combination is the true risk to the servers.

The physical demands of serving appeared to adversely impact the discomfort that developed during the shifts. Relatively, there were increases in pain at the end of the shift as compared to the start in the upper back (by 55%), neck (by 45%), lower back (by 50%), hand/wrist (by 35%), and leg/foot ( by 60%). The increase in the pain for the majority of the body regions appears to be linked to the physical demands of the job such as walking/standing, frequency and weight carried on the trays, and poor postures when handling the trays. One of the more surprising results was the actual decrease in pain in two regions: shoulders (by 45%) and knees (by 30%). This decrease in pain was particularly surprising given the prevalence of holding the trays on shoulders (relating to shoulder pain) and the extensiveness of the standing and walking (relating to knee pain).

There were several limitations to the study that need to be considered when interpreting the results. First, the study was a descriptive cross-sectional study, which limits the causal inference that could be drawn. The observational study was also a preliminary investigation into the demands on the servers in restaurants for a single shift. As such, statistics were limited to descriptive statistics with examples: 1) means per hour and 2) frequencies of events. The study aimed to describe the potential demands, not compare between groups such as types of restaurants, expertise, or other groupings.

Second, the study evaluated a low number of servers (n=20), each with only one shift of observation. The main driver for the low number of participants was the amount of time that the observations required as a member of the research team had to directly follow the server the entire shift. Further, several servers at the restaurants refused to participate, limiting the potential sample population. In the perfect world, the research team would like to have had more subjects from a more diverse group of restaurants.

Third, the number of restaurants was limited to 3 places that had very diverse cuisine and environments. A total of 13 restaurants were contacted with 10 refusing to allow the research team to observe. The main concerns were the potential impact on service and the safety of the food during observation. While methods minimized both of these, these restaurant owners decided not to participate. In general, the results were consistent between the different restaurants as well as between lunch and dinner service, indicating that the results appear to be robust and applicable to the broader restaurant industry. However, the demands, lifting techniques and exposures may be linked to these restaurants and may be different in other facilities.

Fourth, the study only evaluated one shift per person, which neglected variability for daily or seasonal variation. Every attempt to minimize this biased was done by sampling on different days and at different shifts (e.g. lunch vs. dinner). The research team observed over the entire shift, and thus, likely captured an accurate representation of the demands on servers, although they may be under-representative of the typical demands. The perceptions of the servers would support this as many of the servers indicated the observed shift was less demanding than normal.

Fifth, the study population was not diverse with respect to minorities and gender. The entire observed population was Caucasians but we would not expect any difference due to race. Further, the majority of the servers were female (75%) with an average age of 26 years. Thus, the study population was young and relatively experienced (averaged 4.4 years of experience). The younger population may have reflected the type of restaurants, location, or potentially the demographic that is in this industry. A younger workforce likely reflects the typical flexibility and educational level of individuals are servers as oftentimes these jobs are stepping stones to other jobs.

Sixth, the ergonomic stressors were evaluated by observations and not directly measured by sensors or goniometers. It was not feasible to use these more quantitative measures given the safety concerns and time sensitive nature of serving. Observations allowed the research team to not interfere with the servers and elevate all concerns with food safety. Future studies need to investigate actual measurement with devices that can be easily used and eliminate safety concerns. Further, when a study is done, reflecting often allows you to identify things that could have been better measured. One item that the research team has identified as a key item was the table height when the trays where placed on them

Seventh, the study was also evaluated in relation to pain and not actual injuries, which requires long-term assessment. Pain provides a short-term assessment of the negative impact of the stressors. As indicated by Ferguson and Marras [11], musculoskeletal disorders happen in a spectrum starting with pain and continuing to reported injury to disability. Future studies need to look at servers longitudinally to identify the prevalence of injuries and disabilities, thus identifying the long-term effects.

While the study has many concerns with regard to sample size, study design and observation methods, the study was the first step into understanding of demands on servers during their shifts. To date, there has been little research completed investigating the demands and musculoskeletal disorders for servers [27,28]. Given the large number of servers currently employed in the United States, the research provides some insight into the weight lifted and the awkward postures adopted when serving food to the customers. Future research will need to concentrate on better methods to more accurately capture the physical and postural demands for servers in many different types of restaurants. The bottom line is that larger and more robust studies are needed to better understand the ergonomic and physical demands for servers in the vast variety of restaurant environments [33–38].

## Conclusion

The most notable result from the study was amount of weight lifted and carried regularly by servers (16.4 kg per hour or 6.3 kg per tray), which theoretically poses significant risk for the low musculoskeletal problems. However, there are some indications that the observed demands were below expected as more than half of the servers indicated that the observed days were less busy than their typical work days. Thus, the observed number of trays carried may be significantly lower than typically seen for servers impacting the servers more on busier days. Additionally, there was a large portion of the time spent standing or walking with 90% of the servers indicating they stand between 5 and 8 hours per shift. Based on the ActivPAL, servers sat down about 25% of the time which would be in direct contrast to the perceptions that indicated 55% of the servers had less than 1 break per shift. Limited breaks could further enhance the adverse effects of the high physical demands and being constantly on their feet. The most widely used technique to transfer the trays was holding tray directly in front of the server, potentially being the more demanding tray carrying technique. The use of trays or tables would undoubtedly minimize the physical demands on the servers with respect to holding the weight of the tray, food and dishes. The physical demands of serving appeared to adversely impact the discomfort that developed during the shifts. These awkward postures, heavy trays of food, and long periods of time standing may take a toll on the servers. Since this is an initial study investigating the demands of servers, there still needs more research in order to better understand the demands required.

## Figures and Tables

**Table 1:** Demographic data, observation time and restaurant breakdown for the three restaurants (average and standard deviation).

Restaurant	Age (Years)	Standing Height (cm)	Body Weight (kg)	Total Experience (Years)	Current Experience (Months)	Observation Time (Hours/Server)	Total Time Observed (Hours)
1	26.4 (6.8)	168.0 (6.3)	72.8 (25.8)	5.0 (3.0)	14.6 (8.1)	3.8 (0.8)	30.4
2	23.8 (2.9)	170.2 (8.6)	68.7 (20.1)	3.0 (1.6)	15.0 (12.0)	3.1 (0.4)	15.4
3	27.0 (3.5)	169.5 (7.1)	76.3 (20.3)	4.7 (2.2)	22.3 (18.6)	3.3 (0.9)	23.2
ALL	26.0 (4.9)	169.0 (6.9)	73.0 (21.7)	4.4 (2.5)	17.4 (13.3)	3.4 (0.8)	68.9

Restaurant	Gender		TOTAL		Shift Type
	Male	Female	Lunch	Dinner	
1	2	6	8	2	6
2	2	3	5	3	2
3	1	6	7	4	3
ALL	5	15	20	9	11

**Table 2:** Server perception of their typical workload based on Workload Perception Survey.

Would you describe today’s shift as	FREQ	%
Significantly lighter than normal	8	40
Slightly lighter than normal	4	20
Typical of normal	8	40
Slightly heavier than normal	0	0
Significantly heavier than normal	0	0
How many people do you serve per hour on a normal day?	FREQ	%
1 to 3 people	1	5
4 to 6 people	0	0
7 to 9 people	5	25
10 to 12 people	6	30
12 or more people	8	40
On your typical shift, how many tables would you serve per hour?	FREQ	%
1 to 2 tables per hour	1	5
3 to 4 tables per hour	8	40
5 to 6 tables per hour	7	35
7 to 8 tables per hour	0	0
9 or more tables per hour	4	20
On average how many hours a week do you work?	FREQ	%
Less than part time (less than 20 hours a week)	2	10
Part time (20 hours a week)	10	50
Full time (40 hours a week)	7	35
More than 40 hours a week	1	5
On average how long do you stand while working your normal shift?	FREQ	%
Less than an hour	0	0
Greater than an hour but less than 2 hours	0	0
Greater than 2 hours but less than 5 hours	1	5
Greater than 5 hours but less than 8 hours	18	90
Greater than 8 hours	1	5
How often do you get to take a break?	FREQ	%
Once an hour	2	10
Once a shift	8	40
Whenever you want	7	35
Never	3	15
What days do you normally work within a week?	FREQ	%
Mixed	12	60
Only Weekends	1	5
Only Weekdays	3	15
Only Mornings	1	5
Only Nights	3	15

**Table 3:** Amount of weight carried (per hour and per tray) and number of trays as a function of restaurant, carrying style and shift type (average and standard deviation).

Restaurant	Total Weight Per Hour (kg)	Weight Per Tray (kg)	Number of Trays Carried Per Hour (#)
1	13.1 (10.8)	5.7 (2.1)	2.3 (1.5)
2	15.3 (8.3)	7.3 (2.6)	2.2 (0.8)
3	21.2 (12.6)	6.3 (2.4)	3.6 (2.0)
All	16.4 (11.3)	6.3 (2.4)	2.7 (1.7)
Carrying Style			
Combo	15.6 (9.1)	5.5 (2.3)	2.7 (0.6)
Finger	13.2 (13.6)	5.7 (7.4)	1.7 (0.8)
Front	16.8 (12.0)	6.7 (6.5)	3.1 (0.8)
Shoulder	15.2 (3.1)	6.8 (6.0)	1.3 (0.2)
Shift Type			
Lunch	16.3 (12.3)	7.1 (2.5)	2.5 (1.6)
Dinner	16.6 (10.7)	5.8 (2.2)	2.9 (1.7)

**Table 4:** Frequency of the number (percentage of total) of times a tray was placed at table and style of carrying tray.

	Tray Jack at Waist Level	Tray Jack at Thigh Level	Holding Tray	Set on Table	Total
All	14 (10.5%)	9 (6.8%)	26 (19.5%)	84 (63.2%)	133
Shift Type					
Lunch	9 (6.8%)	9 (6.8%)	6 (4.5%)	22 (16.5%)	46
Dinner	5 (3.8%)	0 (0.0%)	20 (15.0%)	62 (46.6%)	87
Restaurant					
1	0 (0.0%)	9 (6.8%)	12 (9.0%)	12 (9.0%)	52
2	14 (10.5%)	0 (0.0%)	5 (3.8%)	5 (3.8%)	24
3	0 (0.0%)	0 (0.0%)	9 (6.8%)	48 (36.1%)	57
Carrying Style					
	In Front	On Shoulder	Flat Palm	Finger Tips	Total
All	93 (69.9%)	23 (17.3%)	10 (7.5%)	7 (5.3%)	133
Shift Type					
Lunch	24 (18.0%)	14 (10.5%)	0 (0.0%)	6 (4.5%)	44
Dinner	69 (51.9%)	9 (6.8%)	10 (7.5%)	1 (0.8%)	89
Restaurant					
1	28 (21.1%)	11 (8.3%)	10 (7.5%)	3 (2.3%)	54
2	12 (9.0%)	8 (6.0%)	0 (0.0%)	4 (3.0%)	24
3	53 (39.8%)	4 (3.0%)	0 (0.0%)	0 (0.0%)	57

**Table 5:** Number (Percentage of total number) of postures identified as awkward during serving.

	Elbow Flexion	Shoulder Flexion	Shoulder Abduction	Wrist Flexion	Wrist Extension	Neck Side Bending	Back Extension	Back Side Bending	Total
**All**	85 (32.7%)	17 (6.5%)	30 (11.5%)	18 (6.9%)	29 (11.2%)	55 (21.2%)	11 (4.2%)	15 (5.8%)	260
**Shift Type**									
**Lunch**	33 (12.7%)	6 (2.3%)	21 (8.1%)	4 (1.5%)	21 (8.1%)	20 (7.7%)	11 (4.2%)	3 (1.2%)	119
**Dinner**	52 (20.0%)	11 (4.2%)	9 (3.5%)	14 (5.4%)	8 (3.1%)	35 (13.5%)	0 (0.0%)	12 (4.6%)	141
Restaurant									
**1**	32 (12.3%)	12 (4.6%)	13 (5.0%)	2 (0.8%)	14 (5.4%)	26 (10.0%)	5 (1.9%)	6 (2.3%)	110
**2**	20 (7.7%)	0 (0.0%)	13 (5.0%)	1 (0.4%)	11 (4.2%)	7 (2.7%)	5 (1.9%)	5 (1.9%)	61
**3**									

**Table 6:** Physical activity for servers working as a function of overall, restaurant, and shift type (average and standard deviation).

	% of Time Sitting	% of Time Standing	% of Time Walking	Total Number of Steps	Energy Expenditure MET▪h
**All**	24.5 (25.5)	44.6 (16.7)	30.9 (13.9)	1018.3 (609.8)	1.5 (0.5)
**Shift Type**					
**Lunch**	15.3 (24.0)	49.4 (15.2)	35.3 (14.8)	1139.5 (609.8)	1.8 (0.4)
**Dinner**	33.6 (24.3)	39.9 (13.3)	26.5 (11.9)	697.1 (421.3)	1.2 (0.5)
**Restaurant**					
**1**	23.6 (25.6)	47.4 (16.7)	29.0 (14.8)	946.7 (606.5)	1.5 (0.5)
**2**	19.2 (23.1)	45.0 (12.0)	35.8 (13.8)	1327.7 (690.3)	1.7 (0.5)
**3**	27.6 (27.8)	42.2 (15.1)	30.3 (13.8)	936.0 (577.8)	1.4 (0.6)
